# Linear IgA Bullous Dermatosis: A Rare Immune-Related Cutaneous Adverse Event Due to Pembrolizumab

**DOI:** 10.7759/cureus.113216

**Published:** 2026-07-23

**Authors:** Lakshya Motwani, Vijay Kumar Doddapaneni, Erin L Heuring

**Affiliations:** 1 Internal Medicine, Mercy Health - St. Vincent Medical Center, Toledo, USA; 2 Dermatology, Mercy Health - St. Vincent Medical Center, Toledo, USA

**Keywords:** immune checkpoint, immune checkpoint inhibitor adverse effects, immune-related adverse event (irae), linear iga bullous dermatosis, pembrolizumab, pembrolizumab cutaneous side effect

## Abstract

Programmed death-1 inhibitor therapy has emerged as a novel modality for various conditions, including solid organ tumors, autoimmune conditions, and other malignancies. The spectrum of their adverse effects continues to evolve as their use expands across different patient populations and for other diseases. Linear IgA bullous dermatosis has not been described as a typical autoimmune bullous condition associated with pembrolizumab therapy. In this study, we describe a rare cutaneous adverse event associated with pembrolizumab therapy. The patient had been receiving pembrolizumab therapy for advanced lung adenocarcinoma for several years. Few cases of linear IgA bullous dermatosis have been documented in association with immune checkpoint inhibitor-related cutaneous adverse events.

## Introduction

Programmed death-1 (PD-1) inhibitors are a class of immune checkpoint inhibitors (ICIs) that allow unchecked activity of cell-mediated immunity against tumor cells [1]. They have been used as targeted treatments for various malignancies, including melanoma, non-small cell lung carcinoma (NSCLC), and cutaneous squamous cell carcinoma. The use of immune checkpoint inhibitors can lead to loss of peripheral tolerance, resulting in a constellation of immune-related adverse events (irAEs). Organ systems primarily affected by the ICIs include the skin, gastrointestinal tract, liver, lungs, and endocrine glands [2]. They can occur in up to 60% of patients on monotherapy with ICI, with 14% of these being high-grade irAEs [2]. Cutaneous irAEs are the most common among these adverse events, including rash, pruritus, and vitiligo [3]. According to a single-center retrospective study, bullous autoimmune conditions associated with ICI occur in approximately 1% of cases and include bullous pemphigoid, linear IgA bullous dermatosis (LABD), and bullous lichenoid dermatitis [4]. LABD is a rare autoimmune blistering condition that involves the skin's basement membrane. It is characterized by subepidermal tense bullae with a distribution pattern that varies with age [5,6]. We describe a case of LABD in a patient after the use of pembrolizumab for non-small-cell adenocarcinoma of the lung.

## Case presentation

**Figure 1:**
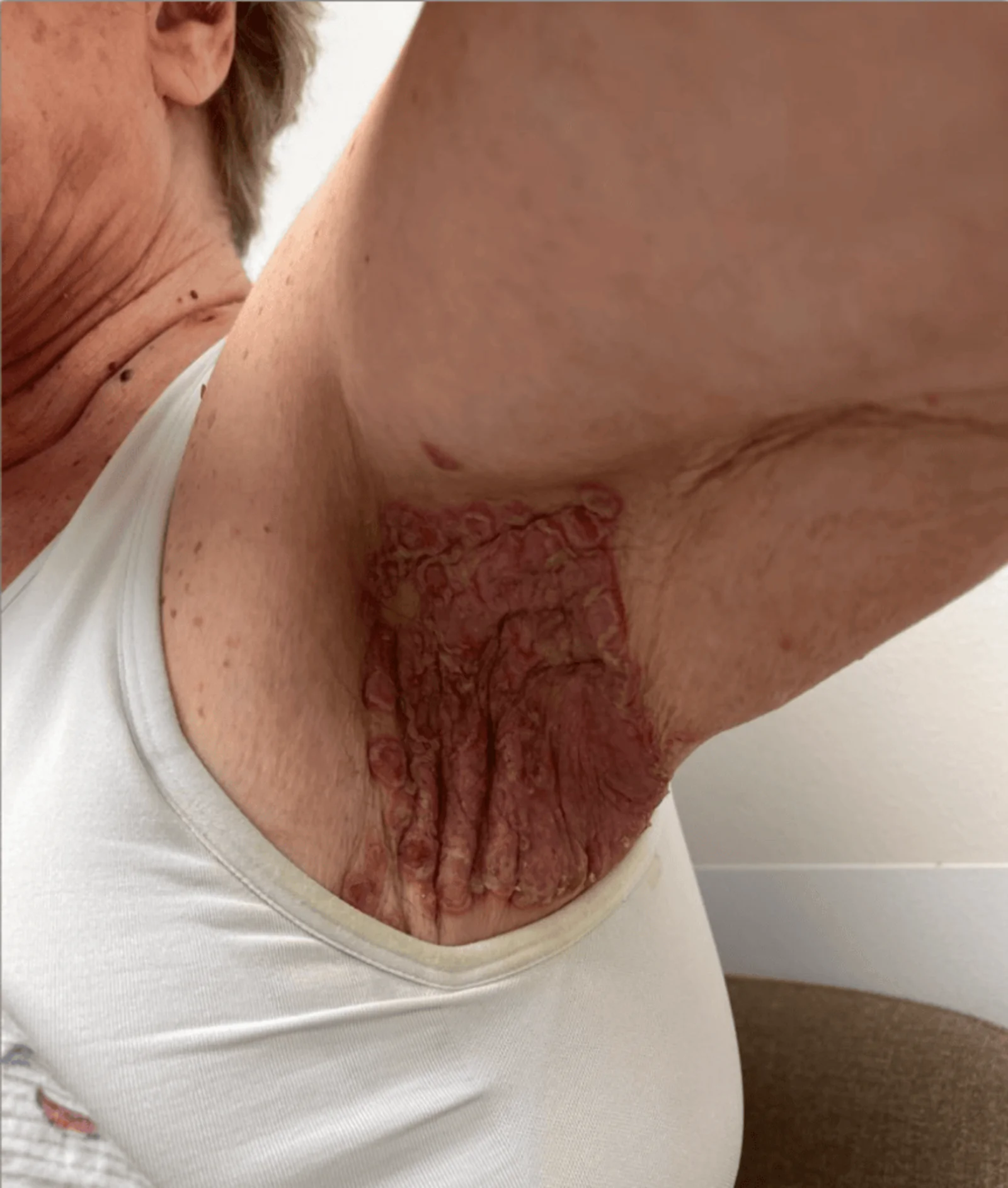
Before topical steroid therapy.

**Figure 2:**
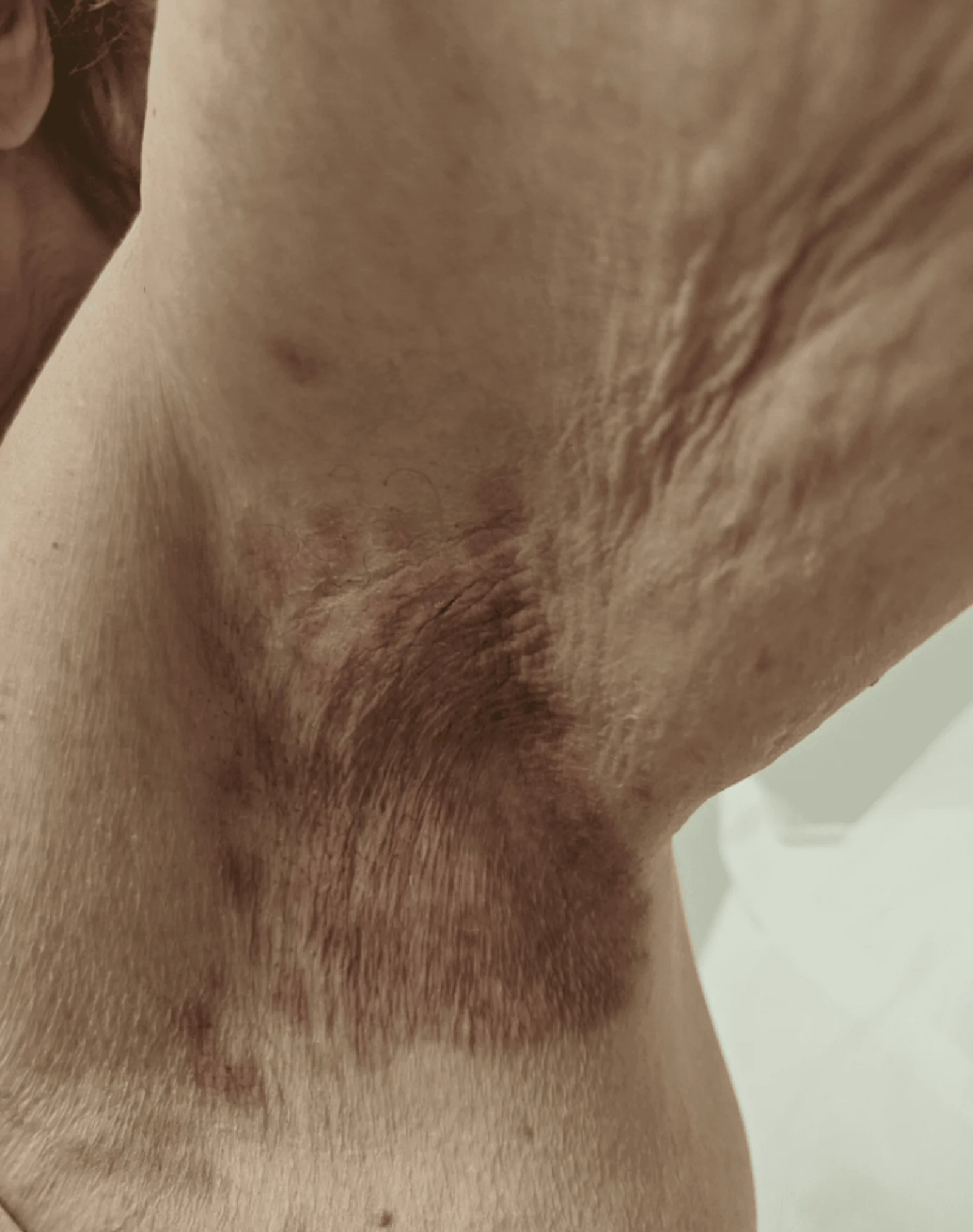
After topical steroid therapy and discontinuation of pembrolizumab.

**Figure 3:**
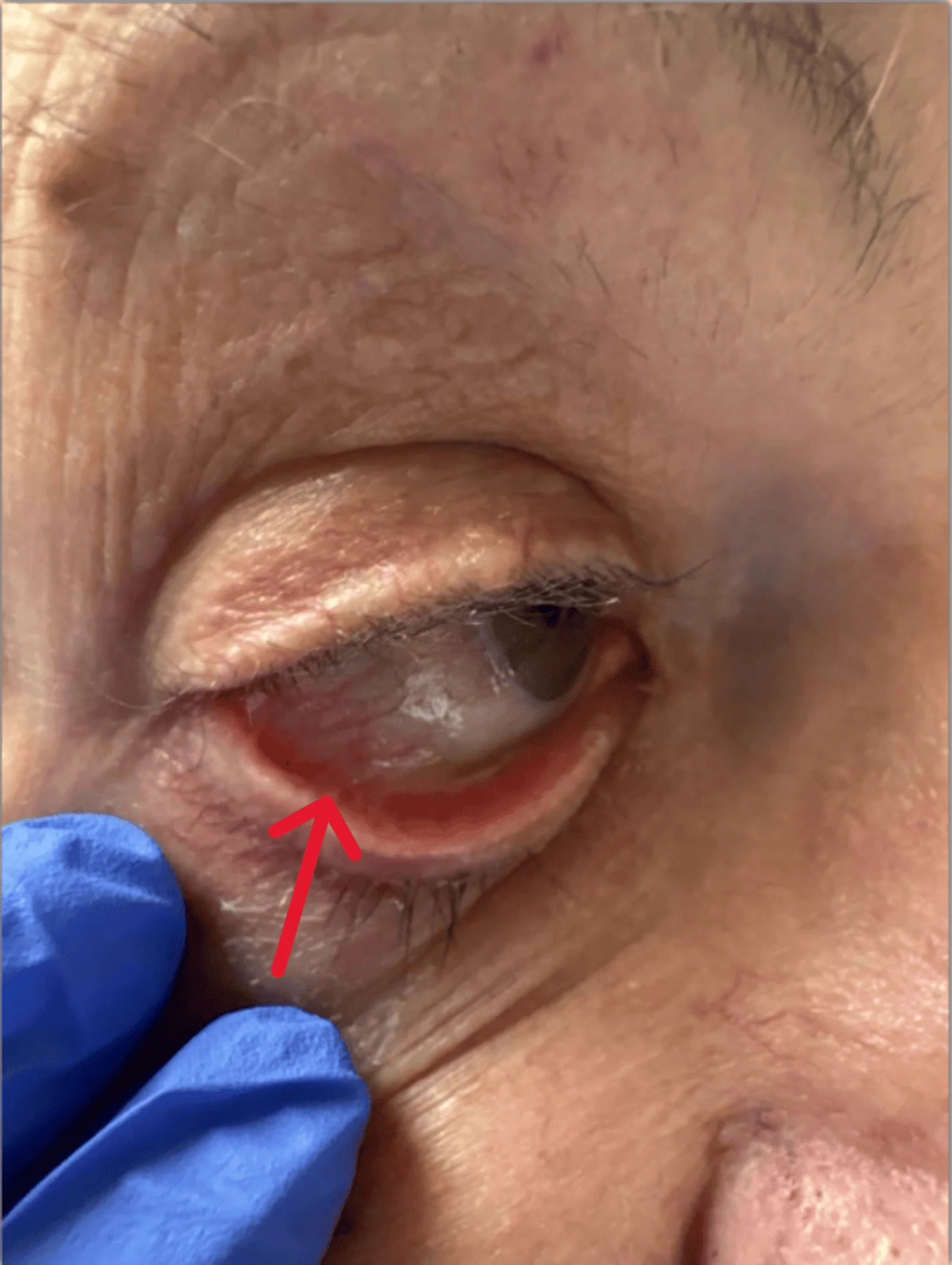
Symblepharon of the right eye (arrow).

A 69-year-old female patient with a history of hypothyroidism, type 2 diabetes mellitus, and hypertension developed a maculopapular rash in the bilateral axilla, groin, neck, and scalp (Figures 1, 2). The rash progressed to painful, pruritic bullae that ruptured. There was involvement of more than 10% of the body surface area. The patient had been diagnosed with primary adenocarcinoma of the lung with metastasis to the chest wall and left gluteal region and was on long-term therapy with pembrolizumab due to high programmed death-ligand 1 (PD-L1) expression in the tumor. The patient had been on pembrolizumab therapy for four years before developing the skin lesions. Therapy with pembrolizumab was initiated every 21 days, and 55 cycles of this treatment had been completed. It is interesting to note that the patient also developed a symblepharon on the right eye for which she received topical steroids and cyclosporine eye drops (Figure 3).

After undergoing a punch biopsy and direct immunofluorescence study, showing subepidermal bullous dermatosis with eosinophils and IgA deposition, suggestive of linear IgA bullous dermatosis (LABD), both IgG and IgA linear deposits were present in the basement membrane. Indirect immunofluorescence showed reactivity consistent with pemphigus and pemphigoid/linear IgA disease. Enzyme assay for BP180, BP230, collagen VII, dsg1, and dsg3 was also negative. A paraneoplastic pemphigoid panel was performed, with negative indirect immunofluorescence staining and a negative IgG envoplakin antibody by enzyme-linked immunosorbent assay (ELISA). LABD was now a confirmed diagnosis.

Dermatological evaluation was done, and the patient was initially treated with a topical steroid and oral antibiotics; however, her skin lesions did not improve on topical therapy alone. Pembrolizumab therapy was also temporarily discontinued for nearly two months, which prevented further progression of the rash. The patient's rash resolved with topical medium- and high-intensity corticosteroid ointments (Figures 1, 2).

After confirmation of the diagnosis, treatment for bullous dermatosis was initiated with dapsone. Dapsone has been shown to benefit patients with autoimmune inflammatory conditions and neutrophilic infiltrates. Dapsone was then switched to colchicine due to a concern for G6PD deficiency-related hemolytic anemia. Maintenance therapy with colchicine in combination with topical steroids stopped the recurrence of the rash, and pembrolizumab therapy was resumed after two months.

## Discussion

Incidence

Immune checkpoint inhibitors (ICIs) have been known to cause various skin reactions during or after treatment. The most common skin eruptions with ICIs are generally vitiligo, pruritus, and maculopapular rash. Common risk factors for developing an immune-related adverse event (irAE) would be treatment for melanoma, prior history of autoimmune skin conditions, and the type of ICI being used for treatment [7,8]. It is interesting to note that CTLA-4 inhibitors have been shown to have a dose-dependent reaction as compared to PD-1/PD-L1 inhibitors. There is no clear relationship between the dose of PD-1/PD-L1 inhibitors and the occurrence of irAEs that has been established [9]. LABD is considered a part of bullous disorders with an autoimmune etiology, the underlying mechanism of which is IgA directed against antigens within the basement membrane zone of the dermo-epidermal junction.

To date, fewer than 10 cases of LABD associated with ICI therapy have been reported in the published literature. The first was described by Jonna et al. in 2019 and included isolated gingival involvement in a patient treated with nivolumab [10]. Two cases of atezolizumab-induced LABD have since been published by Almutairi and Alessa and Aguilar-Duran et al. [11,12]. Momin et al. reported a case associated with combined anti-PD-1 and anti-CTLA-4 therapy in 2023 [13]. Most recently, Saeed et al. reported the only case to date of severe ocular involvement, including cicatricial keratoconjunctivitis and bilateral symblepharon, occurring in the setting of nivolumab therapy [14]. Notably, no prior case of pembrolizumab-induced LABD has been reported in the literature, underscoring the rarity and novelty of the present case.

A linear pattern noted on immunofluorescence with IgA deposits is pathognomonic. Linear IgA disease (LAD) has been shown to exhibit a bimodal age distribution, with cases first observed in children less than five years of age [15]. In their review of available studies, Fortuna and Marinkovich found no evidence of racial or ethnic clusters in LAD [16].

Pathogenesis

The underlying mechanism behind cutaneous irAE with ICIs is via T-cell activation. It is theorized that T cells target antigens at the dermo-epidermal junction because these antigens mimic tumor antigens [17]. LAD is thought to have a combination of humoral and cell-mediated immunity, typically CD4+ T cells that recognize the non-collagenous domain 16A (NC16A) of the BP180 antigen, similar to IgA. Sensitized T-helper cells proliferate and secrete both Th-1 and Th-2 cytokine profiles that, in combination with the IgA autoantibodies, lead to local tissue destruction in the basement membrane zone [18]. Since there is little data explaining the phenomenon of LABD as an irAE with ICI therapy, the underlying molecular basis is poorly understood. Dermatopathological investigations should always be carried out in cases of bullous eruptions for patients undergoing treatment with ICIs.

Clinical grading and management

Eruptions are graded according to body surface area (BSA) involvement as follows: grade 1 (<10% BSA), grade 2 (10-30% BSA), grade 3 (>30% BSA), and grade 4 (life-threatening eruptions requiring intensive care unit or burn unit admission). A multidisciplinary team comprising dermatology, oncology, and immunology specialists is recommended when considering interruption of ICI therapy. Management is based on BSA involvement - topical emollients and corticosteroids for BSA <10%, discontinuation of ICI therapy for BSA >10%, and oral corticosteroids for BSA 10-30%. For patients with BSA >30% requiring hospitalization, prompt initiation of systemic steroids and cessation of ICI therapy are advised [19].

## Conclusions

Linear IgA bullous dermatosis is a rare but important cutaneous immune-related adverse event associated with immune checkpoint inhibitor therapy. This case highlights pembrolizumab as a potential trigger for LABD, including ocular involvement, after long-term treatment. Early recognition and confirmation by histopathology and immunofluorescence are essential for guiding management. Discontinuation of the immune checkpoint inhibitor and initiation of appropriate steroid therapy can lead to clinical improvement and prevent further complications.
